# Pennsylvania Hospital

**Published:** 1849-07

**Authors:** Spenser Sergeant

**Affiliations:** Resident Physician; Pennsylvania Hospital


### PENNSYLVANIA HOSPITAL.—*Surgical Wards.—Service of* DR. PEACE.

### *Cases discharged since May 15th, 1849.*

| | Cured. | By request. | Died. |
|---|---|---|---|
| Abscess, - - - - - - | 1 | 0 | 0 |
| Amputation, - - - - - | 1 | 0 | 1 |
| Burn, - - - - - - | 2 | 0 | 0 |
| Caries, - - - - - - | 0 | 1 | 0 |
| Chronic conjunctivitis, - - | 0 | 1 | 0 |
| Concussion of Brain, - - | 1 | 0 | 0 |
| Contusions, - - - - - | 3 | 0 | 0 |
| Disease of knee joint, - - | 0 | 1 | 0 |
| Dislocations, (simple,) - - | 1 | 1 | 0 |
| " (compound,) - | 0 | 2 | 0 |
| Fractures, simple 7, viz.: | | | |
| Upper jaw, - - - - - | 1 | 0 | 0 |
| Clavicle, - - - - - - | 1 | 0 | 0 |
| Ribs, - - - - - - | 2 | 0 | 0 |
| Humerus, - - - - - | 2 | 0 | 0 |
| Spine, - - - - - - - | 0 | 1 | 0 |

| | Cured. | By request. | Died. |
|---|---|---|---|
| Fractures, compound, 6 viz.: | | | |
| Skull, - - - - - - | 0 | 0 | 2 |
| Fingers, - - - - - | 1 | 0 | 0 |
| Hand, - - - - - - | 1 | 0 | 0 |
| Arm, - - - - - - | 0 | 0 | 1 |
| Thigh and arm. - - | 0 | 0 | 1 |
| Frost-bite, - - - - | 1 | 0 | 0 |
| Gonorrhœa, - - - - | 1 | 0 | 0 |
| Hare-lip, - - - - - - | 0 | 1 | 0 |
| Hemorrhage, - - - - | 1 | 0 | 0 |
| Hernia, (reducible,) - - - | 1 | 0 | 0 |
| Hydrocele, - - - - | 3 | 0 | 0 |
| Inflamed shoulder, - - - | 0 | 1 | 0 |
| " ear, - - - - - | 1 | 0 | 0 |
| " foot, - - - - - | 2 | 0 | 0 |
| Onychia, - - - - - - | 1 | 0 | 0 |
| Paronychia, - - - - | 1 | 0 | 0 |
| Phymosis, - - - - - | 1 | 0 | 0 |
| Prolapsus ani, - - - - | 1 | 0 | 0 |
| Sinus, - - - - - - | 1 | 0 | 0 |
| Stricture of urethra, - - - | 1 | 0 | 0 |
| Syphilis, - - - - - | 3 | 3 | 0 |
| Ulcers, - - - - - - | 5 | 0 | 0 |
| Wounds, - - - - - | 4 | 2 | 0 |
| | 18 | 11 | 5 |

### *Compound Fracture of the Skull. Death from Extravasation of Blood.*

A middle aged man was admitted May 26th, 1849, who was said to have fallen, the night before, from the third story window of a house.

The only external mark of injury was a large, irregular, lacerated wound of the scalp over the left parietal bone.

The scalp was separated from the bone for some distance, but the pericranium was still attached, nor could any fracture be detected.

His skin was cold, pulse at the wrist absent, pupils dilated, and respiration slow. There was no paralysis.

He was stimulated freely, both externally and internally, and, by the afternoon, he rallied so as to tell his name, but would answer no other question intelligibly. Towards evening, however, his breathing became more difficult, he ceased rolling and tossing in the bed, and died comatose, at 10 o'clock.

The Autopsy was made twelve hours after death.

There was a wound, three inches in the longest direction, irregular in shape, on the left side of the head. Great extravasation under the scalp. Pericranium undetached. The calvarium being removed, a fissure was discovered, extending from the protuberance of the left parietal bone down through the petrous portion of the temporal bone to the foramen magnum. The fissure gradually widened as it approached the base of the skull, so that, though immediately beneath the external wound, it was a mere crack, scarcely perceptible, yet, as it approached the base, it gradually became wider, so as to admit a good large probe.

Immediately underneath the fracture there was a large clot upon the dura mater. This membrane was not wounded, but on removing it a further extravasation was found, extending over the greater part of the hemisphere down to the base. The vessels of the meninges were very turgid, and the substance of the brain was very vascular, but of good consistence. There was no rupture of brain, internal clot or bloody effusion into the ventricles.

This man on admission presented the appearance of one suffering rather from severe concussion, than of compression of the brain—doubtless the slow extravasation was going on which caused his death.

It would be interesting to know whether the fracture was caused by violence directly to the cranium, or whether it was the result of counterstroke. That violence was received by the cranium we know from there being a large external wound, but there was no such injury done to the bone, no comminution or depression as one would expect after such a fall. The injury to the bone was greatest at the base, and gradually disappeared above, as though the transmitted force was received there where its effects were most apparent, but as its force became spent, its effects also ceased.

In both of the cases of fractured humerus the internal condyle was the seat of fracture, and the patients were children.

The lateral angular splints generally recommended in these cases are seldom used here now, but in their stead an angular splint, slightly grooved to fit the rotundity of the limb, is applied to the front of the arm. This extends from the fingers to a short distance above the elbow joint, and the angle is changed daily, either by means of a movable joint or by substituting another splint of a different angle.

Two cases of dislocation of the clavicle were discharged during the past month. One, of the acromial extremity upwards, and one of the sternal extremity forwards.

The former, which was caused by a blow from a slung shot on the part, was treated with Fox's apparatus for fractured clavicle, and was discharged in six weeks perfectly well.

The latter was caused by a fall, and was treated with a pad in the axilla, a short sling and a bandage confining the arm tightly to the side.

He was discharged by request before a cure was effected.

The cases of hydrocele were treated as recommended by Mr. Martin formerly of Calcutta: namely, the injection of tincture of iodine which is allowed to remain in the sac. The formula is as follows: tincture of iodine, half a drachm, water, a drachm and a half; to this is added a little alcohol to prevent the precipitation of the iodine.

Dr. Peace has used this in all the cases which have come under his care during the past three months, and invariably with success. In two of the last three cases, the port-wine injection had been used previous to their admission, without preventing a return of the complaint.

SPENSER SERGEANT, M. D. *Resident Physician.*

*Pennsylvania Hospital, June* 15*th* 1849.

